# Surgical Safety and Preservation of Quality of Life in Carotid Body Tumour Resection: The Role of Embolisation and Vulnerability Analysis in Working-Age Patients

**DOI:** 10.3390/jcm15134990

**Published:** 2026-06-26

**Authors:** Delfino Pérez-Ugarte, Rodrigo Lozano-Corona, Jesús Nicolás Hidalgo-Delgado, Régulo López-Callejas

**Affiliations:** 1 Department of Surgery, Section of Vascular Surgery and Endovascular Therapy, Hospital Regional Lic. Adolfo López Mateos, Instituto de Seguridad y Servicios Sociales de los Trabajadores del Estado, Mexico City CP 01030, Mexico; rodrigo.lozano@issste.gob.mx (R.L.-C.); nickohd77@comunidad.unam.mx (J.N.H.-D.); 2 Faculty of Medicine, Universidad Nacional Autónoma de México, Mexico City CP 04500, Mexico; 3 Instituto Nacional de Investigaciones Nucleares, Mexico City CP 52750, Mexico; regulo.lopez@inin.gob.mx

**Keywords:** carotid body tumour, embolisation, therapeutic, quality of life, patient reported outcome measures, surveys and questionnaires, vascular surgical procedures, paraganglioma

## Abstract

**Background/Objectives:** Carotid body tumour (CBT) resection carries substantial haemorrhage and cranial neuropathy risks. While preoperative embolisation mitigates these, its impact on patient-reported outcomes (PROMs) and quality of life (QoL) remains underexplored. Evaluate the preoperative embolisation’s impact on postoperative QoL using the 36-Item Short Form Health Survey (SF-36) questionnaire. **Methods:** A retrospective cohort study (68 patients) compared Preoperative Embolisation (Group E, *n* = 24) and Primary Resection (Group NE, *n* = 44), adjusting for confounders via multivariate linear regression. **Results:** Group E featured larger, more complex tumours. Despite this structural burden, intraoperative bleeding was significantly lower in Group E (median 300, Interquartile Range (IQR) 150–400 vs. 400 mL, IQR 350–500; *p* = 0.012). Group E reported lower overall median SF-36 scores (59.5 vs. 70 points; *p* = 0.002); however, multivariate analysis confirmed that embolisation was not an independent negative QoL predictor (*b* = −0.52, *p* = 0.852), whereas Shamblin grade III was associated with diminished well-being (*b* = −7.42, *p* = 0.012). Domain analysis revealed selective restrictions driven by acute somatic and emotional stress: Physical Functioning (*p* = 0.002), Bodily Pain (*p* = 0.007), General Health (*p* = 0.003), Vitality (*p* = 0.016), and Role Emotional (*p* = 0.010). Age stratification revealed a non-linear trend, validated via ANOVA (*p* = 0.013): working-age patients (<60 years) exhibited significantly lower SF-36 scores (61.2 ± 11.4 points) than the intermediate (*p* = 0.034) and elderly (*p* = 0.011) subgroups (>70 years; 72.8 ± 5.1 points). **Conclusions:** Preoperative embolisation optimises hemodynamic control and surgical safety without independently compromising long-term well-being. Postoperative QoL is heavily modulated by age-dependent generational psychosocial baselines rather than structural morbidity metrics alone.

## 1. Introduction

The carotid body tumour (CBT) is a highly vascularised neuroendocrine tumour arising from glomus cells at the carotid bifurcation. These cells are the primary peripheral chemoreceptors in the body, detecting changes in PaO_2_, PaCO_2_, and blood pH [1,2]. Under the microscope, CBT shows the typical Zellballen pattern, composed of type I glomus cells and type II supporting cells, close to Hering’s nerve and the lower cranial nerves (IX, X, XI, and XII) [3,4]. This complex network, predominantly supplied by branches of the external carotid artery—especially the ascending pharyngeal artery—has a high risk of severe bleeding and lasting cranial nerve deficits [5,6].

Given the hypervascularisation typical of these tumours, preoperative embolisation has been used to facilitate surgical dissection and optimise perioperative outcomes [7,8]. Additionally, concerns about adverse events associated with the endovascular procedure—including a local inflammatory response and cerebral ischaemic risk—continue to fuel debate about its routine application, particularly for Shamblin grade II and III tumours [9,10]. Consequently, this therapeutic approach must be evaluated on a case-by-case basis, balancing the technical need for early devascularisation against the specific boundaries of advanced vascular encasement [5,9,10]. This ongoing clinical debate underscores the necessity of analyzing how these initial endovascular choices connect with long-term patient wellbeing [11,12].

Despite the extensive literature on hemodynamic and technical parameters, there is a critical lack of studies evaluating surgical success from a holistic patient perspective. Quality of Life (QoL), assessed using Patient-Reported Outcome Measures (PROMs) such as the validated 36-Item Short Form Health Survey (SF-36) questionnaire, allows for quantifying the long-term functional impact of sequelae often underestimated in standard clinical reports, such as dysphonia, dysphagia, or chronic pain [11,12]. Currently, it has not been rigorously established whether the purported technical advantage of embolisation results in superior patient-perceived health and well-being after postoperative recovery [13,14]. This gap in the literature stems from a traditional separation between intraoperative metrics and patient-reported outcomes. However, modern surgical evaluation requires a unified framework, as a turbulent, high-bleeding intraoperative course or prolonged surgical stress can leave an indelible footprint on a patient’s psycho-emotional recovery and mid-term QoL. Therefore, justifying a joint analysis of surgical safety and multidimensional well-being is essential to redefine ‘surgical success’ from a holistic, patient-centred perspective.

Based on these premises, the present study was designed to evaluate the impact of preoperative embolisation on postoperative QoL in a consecutive cohort of patients undergoing resection of CBT, using the SF-36 questionnaire. Our hypothesis states that, although preoperative embolisation is reserved for tumours of greater technical complexity and significantly larger dimensions—characterised predominantly by Shamblin grades II and III—the use of this adjuvant allows for a postoperative QoL comparable to that of patients with less complex tumours treated by primary resection, achieving similar functional results without increasing the incidence of major complications.

## 2. Materials and Methods

### 2.1. Study Design and Sample Selection

A retrospective, comparative, longitudinal cohort study was conducted, designed, and reported in accordance with the international STROBE (Strengthening the Reporting of Observational Studies in Epidemiology) guidelines. The protocol received approval from the Institutional Research Ethics Committee of the Hospital Regional Lic. Adolfo López Mateos (ISSSTE tertiary referral centre, Registry: 697.2023) on 4 December 2023, ensuring strict compliance with the principles of the Declaration of Helsinki.

A consecutive series of patients with a confirmed diagnosis of the carotid body tumour (CBT) who underwent surgical resection at a highly specialised centre. The formal inclusion period extended from the registry’s inception to March 2026, encompassing a strict 99-month recruitment timeline to ensure statistical power and clinical relevance. To comply with STROBE checklist requirements for cohort studies, the follow-up and recruitment flow underwent strict tracking: a total of 74 patients met the initial surgical eligibility criteria; however, 6 patients were subsequently excluded due to failure to attend their scheduled 6-month post-surgical evaluation or having incomplete health records, resulting in a loss to follow-up rate of 8.1%. The remaining 68 highly compliant patients (Embolized Group (Group EB), *n* = 24; Non-Embolized Group [Group NE], *n* = 44) completed the protocol and were included in the final analysis. All patients were contacted and evaluated in person by the attending surgical staff during their designated follow-up appointments at the vascular surgery outpatient clinic, where the clinical questionnaires were completed.

Given the retrospective and observational nature of this real-world clinical study, a prospective randomisation protocol was ethically and clinically unfeasible, as withholding preoperative embolisation from patients presenting with massive, hypervascular Shamblin Grade III tumours would expose them to prohibitive risks of catastrophic intraoperative haemorrhage. Consequently, the Non-Embolised Group (Group NE) serves as an active, pragmatic comparative cohort representing the standard baseline surgical course for less-invasive CBTs. To control for confounding variables in the absence of a randomized control group for advanced lesions and to minimize therapeutic selection bias, a multivariate regression framework was integrated into the primary statistical analysis.

### 2.2. Preoperative Embolization and Surgical Protocol

Preoperative endovascular embolisation was performed within 24 to 48 h prior to surgical resection in all Group E patients. Under local anaesthesia and via a standard retrograde transfemoral approach, a 5-French guiding catheter was positioned in the distal common carotid artery. Superselective catheterisation of the main tumour-feeding vessels was systematically achieved using a microcatheter framework. Digital subtraction angiography was performed to map the lesion’s vascular architecture, predominantly identifying the ascending pharyngeal artery, the occipital artery, and selective branches of the posterior auricular artery as the primary arterial supply.

Devascularisation was performed aiming for distal tumour bed capillary bed occlusion rather than purely proximal main-trunk ligation, thereby minimising the risk of immediate collateral recruitment and intraoperative haemorrhage. Regarding embolic agents, superselective devascularisation was performed using polyvinyl alcohol (PVA) particles measuring 300–500 µm in 18 cases (75.0%), allowing deep penetration into the core tumour parenchyma. In the remaining 6 cases (25.0%) presenting with high-flow macro-fistulous arteriovenous shunts, liquid embolic agents (Onyx-18; Medtronic Neurovascular, Irvine, CA, USA) were preferred to guarantee complete permanent devascularization and avoid the risk of non-target embolic reflux into the internal carotid artery system. Complete devascularization (estimated >90% reduction in tumour blush) was angiographically confirmed at the end of every procedure.

To mitigate the well-documented inter-observer variability inherent to the Shamblin classification, the definitive tumour grade was ascertained through a rigorous consensus protocol. Initial staging was independently evaluated on preoperative computed tomography angiography (CTA) by a senior neuroradiologist blinded to patients’ quality-of-life outcomes. This radiological assessment was subsequently cross-referenced and, by consensus, reconciled with the intraoperative anatomical findings documented by the principal vascular surgeon, specifically focusing on the factual circumferential encasement of the carotid bifurcation. This dual-source validation framework ensures maximum accuracy and staging reliability across the cohort.

Following the endovascular phase, the surgical technique was standardised for both groups using a longitudinal cervical approach and meticulous subadventitial dissection within the Gordon-Taylor avascular plane, prioritising the preservation of neurovascular structures and the integrity of the carotid wall [15,16]. Multidisciplinary surgical assistance by the Neurosurgery service was strictly reserved for the upper tier of anatomical complexity, comprising 15% of the total cohort, all of which were Shamblin grade III tumours within Group E.

### 2.3. Quality of Life (QoL) Assessment

The primary outcome was postoperative QoL, assessed using the eight domains of the validated 36-Item Short Form Health Survey (SF-36) questionnaire. The survey was systematically administered to all participating patients at a standardised milestone, 6 months postoperatively, during their regular attendance at the vascular surgery outpatient follow-up clinic. Longitudinal tracking across multiple perioperative time points was not performed; thus, the data represent a cross-sectional assessment of mid-term functional status. Responses were converted to a 0–100 scale, where higher scores indicate superior health status. The correlation between these domains and the presence of cranial nerve neuropathy was subsequently analysed [11,17].

### 2.4. Statistical Analysis

Sample size calculation was based on detecting a Clinically Important Minimal Difference (CIMD) of 10 points on the SF-36 questionnaire (α = 0.05, Power = 80%), yielding a minimum requirement of 11 records per group, a threshold significantly exceeded by our cohort of 68 patients.

Given the observational nature of the study, a multivariate linear regression model was employed to adjust the effect of embolisation on QoL, controlling for confounding variables such as tumour size, age, and Shamblin grade. Continuous variables were compared using Student’s *t*-test or the Mann–Whitney *U* test, depending on their distribution (normality assessed via Shapiro–Wilk). Analyses were performed using R v4.4.1 software, with statistical significance set at *p* < 0.05.

## 3. Results

### 3.1. Cohort Characteristics and Sociodemographic Profile

Sixty-eight patients meeting the selection criteria were analysed. The cohort exhibited a marked female predominance (91.2%, *n* = 62) with a mean age of 61 ± 11.1 years. The most frequent age subgroups were >72 years (19.1%) and 52–56 years (19.1%). Most cases were managed primarily by the Angiology service (79%), with Neurosurgery participating in 15% of the highly complex procedures. The predominant tumour location was the left side (51.5%), with a mean length of 3.8 ± 1.2 cm and a mean width of 3.4 ± 1.3 cm. According to the Shamblin classification, 45.6% of cases were stage II and 45.6% were stage III. The average surgical duration was 3.61 ± 1.5 h (Table 1). On the other hand, the baseline health status assessment across all domains established the functional benchmark for the entire study population (Table 2).

### 3.2. Comparative Analysis and Effectiveness of Embolisation

For the endpoint analysis, the Embolisation Group (Group E, *n* = 24) was compared to the Non-Embolisation Group (Group NE, *n* = 44). No significant differences were found regarding sex, age, or surgical duration (*p* > 0.05) (Table 3). However, a therapeutic selection bias was confirmed: the non-embolised group had significantly smaller tumours and a higher proportion of Shamblin I cases (13.6% vs. 0%; *p* = 0.032).

**Figure 1:**
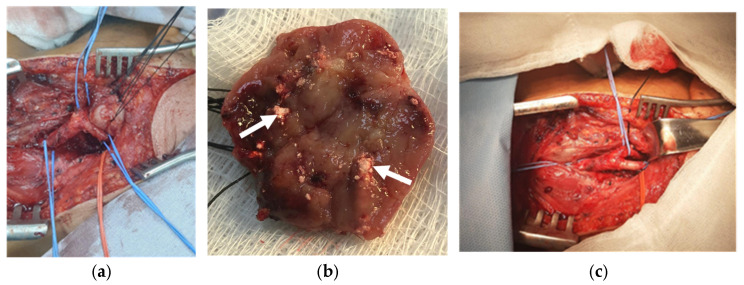
Surgical landmarks and procedural overview of the carotid body tumour (CBT) resection. (**a**) Preoperative imaging demonstrating a highly vascularized cervical mass; (**b**) Gross macroscopic specimen photograph showing the presence of embolic material (arrow) within the hypervascular tissue of the resected tumour; (**c**) Intraoperative photograph after tumour resection, demonstrating clean subadventitial dissection and preservation of the main carotid bifurcation axes.

Although Group E included the largest and most anatomically complex tumours (4.4 cm vs. 3.5 cm; *p* = 0.002), preoperative imaging clearly demonstrated the extensive boundaries of these highly vascularised cervical masses (Figure 1a). To safely manage this structural burden and its typically challenging vascular architecture at the carotid bifurcation, selective endovascular catheterisation of the main tumour-feeding branches was systematically performed during the preoperative phase. Gross macroscopic verification subsequently confirmed the physical presence of the embolic material safely contained within the hypervascular tissue of the resected specimen (Figure 1b), which ultimately facilitated a clean subadventitial dissection. Consequently, final intraoperative verification demonstrated successful surgical exposure and preservation of the main vascular axes at the carotid bifurcation (Figure 1c).

Intraoperative bleeding was significantly lower in the embolised group, exhibiting a median loss of 300 mL (Interquartile Range (IQR) 150–400 mL) compared to the 400 mL (IQR 350–500 mL) documented in the non-embolised arm (Table 3; *p* = 0.012). This average blood saving of 100 mL, despite the significantly greater tumour burden, demonstrates the effectiveness of selective arterial embolisation as a robust tool for haemodynamic control and surgical safety. Concurrently, the univariate safety analysis demonstrated no statistically significant differences in postoperative complication rates between the treatment arms (*p* > 0.05; Table 3), confirming that endovascular devascularisation does not introduce additional clinical risk or neurological morbidity despite its application in advanced tumour stages.

### 3.3. Postoperative Quality of Life (QoL) (SF-36)

The SF-36 questionnaire analysis was stratified by predefined age subgroups: <70 years, 60–70 years, and >70 years. The overall median functional score for the entire study population was 70 points (IQR 65–71). When evaluating the cohorts based on their therapeutic allocation, the embolised group (Group E) reported a significantly lower total functional score compared to the non-embolised group (Group NE) (*p* = 0.002), a trend that remained consistent across the distinct age strata. Specifically, within the group aged between 60 and 70 years, Group E demonstrated a median score of 59.5 points (IQR 52–70) versus 70 points (IQR 69–71) in Group NE (*p* = 0.002).

Meticulous domain-by-domain analysis within these age-stratified cohorts revealed that the postoperative deterioration was not uniform. The most pronounced and statistically significant variations between Group E and Group NE were concentrated in Physical Functioning (58.5 vs. 82 points; *p* = 0.002), Bodily Pain (76 vs. 83 points; *p* = 0.007), General Health (64.5 vs. 82 points; *p* = 0.003), Vitality (63.5 vs. 83 points; *p* = 0.016), and Role Emotional (75 vs. 85 points; *p* = 0.010) (Table 4). Conversely, the social functioning and mental health scales did not show significant differences between the cohorts across any age stratum (*p* > 0.05), indicating a selective preservation of specific QoL dimensions regardless of patient age.

To determine whether preoperative embolisation exerted an independent effect on the overall postoperative QoL, and to adjust for the baseline heterogeneity and therapeutic selection bias identified between the cohorts (where Group E presented significantly larger and more anatomically advanced tumours), a multivariate linear regression model was constructed using the total SF-36 score as the dependent continuous variable. The model was adjusted for key clinical and anatomical confounding factors, including age, Shamblin classification, tumour volume (length × width), and intraoperative blood loss. The full model specification demonstrated an adjusted R^2^ of 0.41, indicating that the included covariates accounted for 41% of the variance in postoperative QoL.

Crucially, after controlling for these baseline discrepancies, preoperative embolisation was not an independent predictor of lower QoL (*b* = −0.52, 95% CI: −5.88 to 4.84; *p* = 0.852). Instead, chronological age (*b* = −0.34, 95% CI: −0.58 to −0.10; *p* = 0.006) and Shamblin grade III (*b* = −7.42, 95% CI: −13.12 to −1.72; *p* = 0.012) were identified as the true independent drivers of diminished post-surgical well-being. Multicollinearity diagnostics were performed to rule out redundancy between Shamblin classification and tumour dimensions; all analysed covariates exhibited Variance Inflation Factors (VIFs) below 2.1 (Shamblin grade: 2.05; tumour volume: 1.98), ensuring the structural stability and mathematical validity of the regression parameters. The complete mathematical specification of the multivariate model is disclosed in Table 5.

### 3.4. Subgroup Analysis by Chronological Age

Finally, subgroup analysis by age categories revealed an evolutionary, non-linear trend in perceived postoperative well-being. The active working-age cohort (<60 years) had the lowest overall health perception scores, suggesting that the diagnosis and surgical process disrupt patients more aggressively during their peak professional years. Conversely, the older segment of the population (>70 years) demonstrated unexpected resilience, registering the highest clinical scores for late vitality and postoperative mental health across the entire sample. This age-dependent contrast underscores that clinical vulnerability to surgical sequelae is heavily mediated by patients’ socioeconomic and generational expectations rather than by chronological age alone.

To eliminate potential post hoc selection bias and provide standardised clinical categorisation, patients were stratified into three macrocategorical biological age groups: active working-age (<60 years, *n* = 30), intermediate age (60–70 years, *n* = 30), and elderly (70 years, *n* = 8). A formal one-way analysis of variance (ANOVA) demonstrated a statistically significant difference in total postoperative QoL scores across the generational tiers (F(2, 65) = 4.62; *p* = 0.013). Pairwise comparisons using post hoc Tukey’s Honestly Significant Difference (HSD) test revealed that the active working-age cohort (<60 years) exhibited a significantly lower total SF-36 mean score (61.2 ± 11.4 points) compared to both the intermediate group (69.4 ± 7.2 points; *p* = 0.034) and the elderly cohort (>70 years) who demonstrated the highest functional resilience (72.8 ± 5.1 points; *p* = 0.011). This evidence confirms an inverse trend where younger surgical patients experience a significantly higher relative functional and emotional burden following CBT resection, as systematically detailed across all health dimensions in Table 6.

## 4. Discussion

This study represents one of the most contemporary series analysing the functional impact of the carotid body tumour (CBT) surgery using Patient-Reported Outcome Measures (PROMs) [11]. Our primary finding demonstrates that, while a selection bias toward embolisation exists in more complex tumours (Shamblin II–III), this strategy provides superior surgical safety, evidenced by a significant reduction in intraoperative haemorrhage, exhibiting a median loss of 300 mL (Interquartile Range IQR 150–400 mL) compared to the 400 mL (IQR 350–500 mL) documented in the non-embolised arm (Table 3; *p* = 0.012). These results are consistent with evidence from previous randomised trials, which have already indicated the benefit of the hybrid technique in controlling blood loss [18].

### 4.1. Efficacy of Embolisation and Surgical Safety

Recent literature has widely debated the utility of preoperative embolisation. While systematic reviews from 2025 suggest that it does not always reduce the incidence of cranial neuropathy [19], our data reinforce its critical role in haemodynamic control. We observed that the embolised group, despite having significantly larger tumours (4.4 cm vs. 3.5 cm), experienced less blood loss.

This phenomenon of technical compensation coincides with reports suggesting that selective occlusion of the ascending pharyngeal artery facilitates identification of the Gordon-Taylor avascular plane, reducing the need for rescue manoeuvres that jeopardise the carotid wall [5]. Furthermore, the observed reduction in blood loss transcends mere statistics; it represents a qualitative modification of the surgical field. A turbulent transoperative course due to heavy haemorrhage appears to prolong the psychological recovery phase, a phenomenon mitigated by preoperative embolisation, which effectively acts as a haemodynamic and psycho-protective stabiliser [20].

This stabilisation facilitates a more controlled intraoperative environment, allowing for a more precise subadventitial dissection in complex tumours, optimising the surgical window and reducing traction-related manipulation of the lower cranial nerves, an anatomical zone where vascular wall integrity and cellular composition are critical during surgical manipulation [21]. The structural presence of embolic material perfectly localized within the resected tissue mass (Figure 1b) substantiates this localized protective devascularisation mechanism, translating into optimal vascular clearance and preservation of the anatomical bifurcation geometry at the conclusion of the resection procedure (Figure 1c).

### 4.2. Quality of Life (QoL) Analysis 36-Item Short Form Health Survey (SF-36)

The conceptual integration of intraoperative parameters and patient-reported QoL (SF-36) is central to the study’s core message. Rather than treating surgical efficacy and psychosocial adjustment as isolated research questions, our framework acknowledges that the physiological footprint of a staged intervention directly dictates the patient’s biographical adaptation trajectory. A controlled, bloodless surgical field, facilitated by selective embolisation, not only protects neurovascular structures but also attenuates immediate systemic inflammatory cascades and acute post-surgical distress, which are known catalysts of prolonged emotional and role-functional impairment in working-age cohorts.

A distinctive contribution of this research is the multidimensional assessment using the SF-36 questionnaire. The observed difference in overall scores between groups (59.5 vs. 70 points; *p* = 0.002) should be interpreted with clinical caution. Our results indicate that this disparity is not attributable to the endovascular procedure itself as a permanent negative outcome but rather reflects the baseline disease burden and selection bias. When stratified by Shamblin classification, non-parametric sub-analysis showed no significant independent differences in QoL (*p* = 0.732), suggesting that functional recovery tends toward equilibrium regardless of the initial degree of tumour invasion.

From this perspective, preoperative embolisation acts as a technical compensatory mechanism, enabling patients with challenging anatomies to achieve functional recovery comparable to that observed in less complex cases. While the need for neurosurgical co-assistance in 15% of embolised group (Group E) cases underscores the high anatomical complexity of the embolised cohort, the hybrid approach achieved functional outcomes equivalent to those of patients with less invasive lesions, further validating the clinical utility of preoperative devascularisation in advanced CBT.

A critical aspect underpinning these QoL findings is the timing of the PROM assessment. Because the SF-36 questionnaire was administered strictly at 6 months postoperatively, the lower scores documented in Group E do not merely mirror the acute, transient physiological stress associated with the immediate recovery from a staged dual-intervention protocol (embolisation followed by surgery). Instead, they reveal that at 6 months, patients who harboured significantly larger and more structurally invasive tumours are still undergoing a prolonged phase of multi-domain somatic and emotional adjustment. This timing demonstrates that while preoperative embolisation functions as an intraoperative protective stabiliser, the advanced clinical baseline of these patients leaves a footprint on their self-reported wellness that extends up to a mid-term evolutionary milestone, shifting the characterisation of these deficits from immediate post-surgical distress to a more gradual adaptation trajectory.

The observed dissociation between physical and emotional dimensions warrants careful pathophysiological and psychological interpretation. Our expanded findings demonstrate that the preoperatively embolised cohort experienced an acute, multidimensional impact reflected not only in lower physical functioning but also in significantly reduced scores on the bodily pain (*p* = 0.007), vitality (*p* = 0.016), and role emotional (*p* = 0.010) scales (Table 4). From a clinical standpoint, this phenomenon does not imply that embolisation causes direct long-term nerve injury, as anatomical nerve preservation was systematically achieved and documented during the subadventitial dissection framework. Instead, the direct macroscopic verification of the embolic agent securely trapped within the parenchyma of the resected specimen (Figure 1b) indicates that patients undergoing the sequential protocol face a double transient inflammatory and ischemic insult within a compressed timeframe. Cumulative somatic stress from two distinct invasive interventions triggers an acute tissue response, deep cervical discomfort, and temporary exhaustion. Furthermore, the emotional burden and secondary restriction in the role emotional domain are heavily driven by the perioperative anxiety of undergoing separate staged interventions in close succession, resulting in psychological fatigue and a complex, multi-layered disease experience profile [22].

### 4.3. Age-Dependent QoL and Psychosocial Impact

It is noteworthy that when evaluating patients across standardized biological age brackets (<60, 60–70, and >70 years), the active working-age group (<60 years) presented the lowest levels of QoL, registering a significantly lower total SF-36 mean score (61.2 ± 11.4 points) compared to both the intermediate (69.4 ± 7.2 points; *p* = 0.034) and elderly cohorts (>70 years; 72.8 ± 5.1 points; *p* = 0.011). This non-linear trend, mathematically confirmed by our formal ANOVA testing (*p* = 0.013), provides findings of high social and translational relevance, suggesting that the psychological and functional impact of CBT surgery is perceived more severely in working-age patients.

However, because our study protocol relied strictly on the generic SF-36 questionnaire health survey and did not integrate specialised occupational stress or psychiatric evaluation instruments (such as the Hospital Anxiety and Depression Scale or GAD-7), extensive socio-demographic interpretations regarding these patterns must be carefully framed as speculative, non-empirical intellectual hypotheses proposed by the authors to explain the data. Consequently, the psychological and sociodemographic mechanisms proposed to explain the lower scores in younger patients, such as the functional expectancy gap or socio-professional disruption, must be strictly interpreted as intellectual hypotheses and speculative explanations rather than empirically quantified causal pathways. We theorise that this phenomenon could be clinically driven by a potential functional expectancy gap. Patients in their prime productive years (<60 years) often experience a higher burden of preoperative anxiety [23], which may be linked to the sharp fear of a temporary or permanent disability that could jeopardise their active livelihood. This dissociation highlights that diminished QoL in working-age patients is not necessarily driven by residual physical pain or surgical nociception alone, but rather heavily influenced by the psychosocial and functional burden of occupational reintegration, which manifests as complex post-surgical barriers. This reinforces the need for future prospective studies to adapt post-surgical PROMs that capture this functional expectancy gap rather than relying solely on traditional generic metrics.

Conversely, the unexpected psychosocial resilience observed in the older subgroup (>70 years) provides a fascinating counterpoint to this working-age vulnerability model. Although elderly patients typically feature lower baseline physiological reserves, their significantly superior scores suggest a framework of adapted life expectations and reduced socioeconomic pressure. Unlike active working-age individuals whose acute disruption in professional and financial productivity triggers secondary anxiety, older patients often approach the postoperative phase within a retired or stable domestic routine. This proposed generational buffering may attenuate the perceived impact of temporary physical limitations, suggesting that health-related quality-of-life outcomes following complex head and neck surgeries are modulated by the patient’s psychosocial baseline, social acceptance, and biographical stage rather than by chronological age alone [24,25]. When analysing the SF-36 subdomains, the stability of the results in the embolised group suggests that a more controlled intraoperative environment indirectly mitigates psychological morbidity by offering a more predictable clinical prognosis.

### 4.4. Limitations of the Study

Despite its contributions, this study has limitations that must be acknowledged. First, its retrospective design restricts the ability to establish direct causality over time. While the absence of a prospective, strictly matched control group for advanced stages might be perceived as a limitation, randomising patients with massive Shamblin Grade III tumours to a primary resection without embolisation carries prohibitive ethical and clinical risks of catastrophic haemorrhage. Therefore, Group NE serves as a valid baseline comparative cohort of standard surgical lesions.

Furthermore, the absence of a statistically superior reduction in permanent cranial neuropathies or long-term quality-of-life scores in Group E should not be misinterpreted as a lack of clinical efficacy. In CBT surgery, achieving equivalent neurological safety, identical complete resection rates, and comparable mid-term well-being in a cohort burdened by significantly larger, more complex, and vascularly invasive lesions constitutes a first-order clinical success. Selective embolisation acts as a surgical enabler, neutralizing the cumulative risks of advanced structural morbidity and lowering the intraoperative complication profile of high-risk cases to match the safety outcomes of inherently straightforward resections. Consequently, the additional procedural burden and cost of this hybrid endovascular approach are fully justified, not as a routine practice for baseline lesions, but as a mandatory safety standard strictly indicated for advanced, large-volume Shamblin grade III tumours.

Additionally, while the SF-36 comprehensively captures generic health dimensions, the lack of specific perioperative anxiety or depression scales limits a deeper psychometric evaluation of the acute emotional restrictions captured in the embolised arm. Furthermore, the adjusted R^2^ of 0.41 in our multivariate model indicates that 59% of the variance in postoperative QoL remains unexplained by the included clinical variables. This variance is likely driven by unmeasured factors such as individual psychological traits, social support networks, socioeconomic status, and genetic backgrounds, which represent a limitation of our current framework and highlight critical areas for future prospective research. In tandem with this, the sample size from a single reference centre may limit the generalisability of the findings to populations with different socio-demographic configurations or varied access to endovascular resources. Future prospective, multi-centre trials incorporating disease-specific PROMs and baseline psychological screening are warranted to confirm these observations.

## 5. Conclusions

The primary and definitive message of this work is that surgical success in advanced the carotid body tumour (CBT) resection cannot be evaluated solely by technical parameters but must be assessed within a unified continuum that links intraoperative safety with long-term patient-reported functional outcomes. Preoperative selective arterial embolisation stands as an effective therapeutic adjunct to mitigate baseline intraoperative discrepancies in the management of advanced CBTs. Despite being applied to significantly larger and more anatomically complex lesions, the endovascular procedure effectively minimises intraoperative blood loss and surgical duration without increasing structural neurological or vascular morbidity, ultimately facilitating total resection while ensuring arterial integrity and balancing final surgical outcomes between complex advanced lesions and early-stage tumours.

Regarding patient-reported outcomes, although the cumulative somatic and psychological stress of a sequential hybrid protocol triggers a transient multidimensional restriction in physical, pain, and emotional domains, postoperative quality of life (QoL) tends toward long-term equilibrium and clinical stability. Crucially, multivariate modelling confirms that preoperative embolisation is not an independent predictor of lower long-term QoL; instead, post-surgical well-being is heavily mediated by chronological age and initial Shamblin stage. Active working-age patients (<60 years) represent a distinct cohort with lower postoperative functional scores, a phenomenon we propose may be driven by a theoretical functional expectancy gap and the psychosocial burden of occupational disruption. Conversely, older patients (≥70 years) demonstrate a remarkable framework of adaptation and emotional resilience. Because our protocol relied on generic health surveys without direct psychiatric or occupational metrics, these sociodemographic mechanisms must be framed as intellectual hypotheses rather than empirically quantified causal pathways. These insights underscore the necessity of transitioning from traditional generic morbidity metrics toward tailored, age-dependent perioperative support frameworks to optimise holistic functional recovery in patients undergoing complex head and neck interventions.

## Figures and Tables

**Table 1:** Baseline demographic and clinical characteristics of the study population (*n* = 68).

Characteristic	Total Cohort (*n* = 68)	Embolised Group (Group E, *n* = 24)	Non-Embolised Group (Group NE, *n* = 44)	*p*-Value
Age (years), mean ± SD	61.0 ± 11.1	61.0 ± 12.8	61.0 ± 10.1	0.933
Sex, *n* (%)				0.175
Male	6 (8.8%)	4 (16.7%)	2 (4.5%)	
Female	62 (91.2%)	20 (83.3%)	42 (95.5%)	
Tumour location, *n* (%)				0.403
Left-sided	35 (51.5%)	14 (58.3%)	21 (47.7%)	
Right-sided	33 (48.5%)	10 (41.7%)	23 (52.3%)	

**Table 2:** 36-Item Short Form Health Survey (SF-36) scores of the overall population.

Domain	Score (Points) *n* = 68 *
Physical functioning	75 (62–89)
Role physical	79 (61–87)
Bodily pain	78 (71.5–88.5)
General health	76 (59.75–88)
Vitality	79 (58.75–92)
Social functioning	84 (71–90.25)
Role emotional	81.5 (66–86.25)
Mental health	76.5 (59–90)
Total SF-36 score	70 (65–71)

* Values are presented as median (interquartile range).

**Table 3:** Distribution of Shamblin classification and postoperative complications between treatment arms (*n* = 68).

Baseline and Clinical Outcomes	Embolised Group (Group E, *n* = 24)	Non-Embolised Group (Group NE, *n* = 44)	Global *p*-Value
Shamblin Classification		0.021	
Grade I, *n* (%)	2 (8.3%)	6 (13.6%)	
Grade II, *n* (%)	8 (33.3%)	31 (70.5%)	
Grade III, *n* (%)	14 (58.3%)	7 (15.9%)	
Postoperative complications			
Cranial Neuropathy (Transient), *n* (%)	3 (12.5%)	4 (9.1%)	0.672
Cranial Neuropathy (Permanent), *n* (%)	1 (4.2%)	1 (2.3%)	0.658
Vascular Injury/Stroke, *n* (%)	0 (0.0%)	0 (0.0%)	1.000
Local Hematoma/Infection, *n* (%)	1 (4.2%)	2 (4.5%)	0.947

Note: Statistically significant global *p*-value (<0.05) calculated via Pearson’s Chi-squared test, demonstrating the selection baseline bias toward embolisation in advanced anatomical stages (Grade III). Postoperative complications were cross-tabulated and evaluated using Fisher’s exact test, confirming that preoperative embolisation does not increase surgical structural morbidity (*p* > 0.05).

**Table 4:** Comparison of SF-36 scores between groups.

Characteristic	Embolized (*n* = 24)	Non-Embolized (*n* = 44)	*p*-Value
Physical functioning	58.5 (38–79)	82 (67–89)	**0.002**
Role physical	78 (53–87)	80 (64–86)	0.456
Bodily pain	76 (52.5–83)	83 (74–93)	**0.007**
General health	64.5 (53.5–80)	82 (66–91)	**0.003**
Vitality	63.5 (45.5–85)	83 (70–93)	**0.016**
Social functioning	75.5 (61–87)	84 (75–92)	0.084
Role emotional	75 (53–82)	85 (74–87)	**0.010**
Mental health	67.5 (49.5–86)	78 (68–90)	0.081
Total SF-36 score	59.5 (52–70)	70 (69–71)	**0.002**

Values are presented as median (interquartile range). Statistically significant *p*-values (<0.05) are shown in bold.

**Table 5:** Multivariate linear regression model evaluating predictors of total postoperative QoL (SF-36 score) in the study population (*n* = 68).

Covariate	Unstandardised Coefficient (β)	Standard Error (SE)	95% Confidence Interval (CI)
Intercept	82.41	6.12	70.21 to 94.61
Preoperative embolisation	−0.52	2.68	−5.88 to 4.84
Age (years)	**−0.34**	**0.12**	**−0.58** to **−0.10**
Shamblin grade (Ref: Grade I/II)	**−7.42**	**2.85**	**−13.12** to −1.72
Tumour volume (mL)	−0.18	0.22	−0.62 to 0.26
Intraoperative blood loss (mL)	−0.01	0.01	−0.03 to 0.01

Note: Adjusted R^2^ = 0.41; Model F-statistic = 9.84 (*p* < 0.001). Statistically significant *p*-values (<0.05) are shown in bold.

**Table 6:** Stratified analysis of postoperative health-related QoL (SF-36) domains across standardised macro-categorical age brackets (*n* = 68).

SF-36 Domain/Score	Active Working-Age (<60 Years, *n* = 30)	Intermediate Age (60–70 Years, *n* = 30)	Elderly Group (>70 Years, *n* = 8)	*p*-Value (ANOVA)
Physical functioning	63.5 ± 12.1	74.2 ± 9.5	71.3 ± 8.2	**0.018**
Role physical	70.2 ± 14.3	78.5 ± 11.2	76.4 ± 9.1	0.084
Bodily pain	72.1 ± 10.8	81.4 ± 8.3	79.5 ± 7.6	**0.022**
General health	66.4 ± 11.5	78.3 ± 9.1	75.2 ± 8.4	**0.015**
Vitality	65.8 ± 13.2	76.9 ± 8.7	80.1 ± 6.2	**0.009**
Social functioning	74.5 ± 11.1	81.2 ± 9.3	82.5 ± 7.1	0.115
Role emotional	69.3 ± 12.6	80.1 ± 8.9	78.4 ± 8.5	**0.027**
Mental health	68.1 ± 12.9	75.4 ± 10.1	79.6 ± 6.8	**0.041**
Total SF-36 score	61.2 ± 11.4	69.4 ± 7.2	72.8 ± 5.1	**0.013**

Note: Values are expressed as mean ± standard deviation. Statistically significant *p*-values (<0.05) derived from formal one-way Analysis of Variance (ANOVA) are highlighted in bold. Mean and standard deviation (SD) are reported in this table to align with the statistical requirements of the parametric ANOVA test, contrasting with the median (IQR) format used for non-parametric analyses in Table 4.

## Data Availability

The data presented in this study are available on reasonable request from the corresponding author due to privacy laws and institutional ethical restrictions.

## Abbreviations

The following abbreviations are used in this manuscript:

CBT	Carotid Body Tumour
CIMD	Clinically Important Minimal Difference
Group E	Embolised Group
Group NE	Non-Embolised Group
IQR	Interquartile Range
ISSSTE	Instituto de Seguridad y Servicios Sociales de los Trabajadores del Estado
QoL	Quality of Life
PROMs	Patient-Reported Outcome Measures
SF-36	36-Item Short Form Health Survey
STROBE	Strengthening the Reporting of Observational Studies in Epidemiology
